# Influence of the ABC Transporter YtrBCDEF of *Bacillus subtilis* on Competence, Biofilm Formation and Cell Wall Thickness

**DOI:** 10.3389/fmicb.2021.587035

**Published:** 2021-04-08

**Authors:** Martin Benda, Lisa Maria Schulz, Jörg Stülke, Jeanine Rismondo

**Affiliations:** Department of General Microbiology, Institute of Microbiology and Genetics, Georg-August University Göttingen, Göttingen, Germany

**Keywords:** genetic competence, biofilm formation, ABC transporter, cell wall homeostasis, *Bacillus subtilis*

## Abstract

*Bacillus subtilis* develops genetic competence for the uptake of foreign DNA when cells enter stationary phase and a high cell density is reached. These signals are integrated by the competence transcription factor ComK, which is subject to transcriptional, post-transcriptional and post-translational regulation. Many proteins are involved in the development of competence, both to control ComK activity and to mediate DNA uptake. However, for many proteins, the precise function they play in competence development is unknown. In this study, we assessed whether proteins required for genetic transformation play a role in the activation of ComK or rather act downstream of competence gene expression. While these possibilities could be distinguished for most of the tested factors, we assume that two proteins, PNPase and the transcription factor YtrA, are required both for full ComK activity and for the downstream processes of DNA uptake and integration. Further analyses of the role of the transcription factor YtrA for the competence development revealed that the overexpression of the YtrBCDEF ABC transporter in the *ytrA* mutant causes the loss of genetic competence. Moreover, overexpression of this ABC transporter also affects biofilm formation. Since the *ytrGABCDEF* operon is naturally induced by cell wall-targeting antibiotics, we tested the cell wall properties upon overexpression of the ABC transporter and observed an increased thickness of the cell wall. The composition and properties of the cell wall are important for competence development and biofilm formation, suggesting that the observed phenotypes are the result of the increased cell wall thickness as an outcome of YtrBCDEF overexpression.

## Introduction

The Gram-positive model bacterium *Bacillus subtilis* has evolved many different ways to survive harsh environmental conditions, i.e., it can form highly resistant spores, secrete toxins to kill and cannibalize neighboring cells, form resistant macroscopic biofilms or become competent for transformation [reviewed in López and Kolter (2010)].

Development of genetic competence is a strategy, which allows bacterial cells to take up foreign DNA from the environment in order to increase the genetic variability of the population. Competence is developed during the transition from exponential to stationary phase of growth as a response to increased cell density and nutrient limitation. In *B. subtilis*, genetic competence is developed in a bistable manner, meaning that only about 10–20% of the cells of a population change their physiological characteristics and become competent for transformation, leaving the rest of the population non-competent (Haijema et al., 2001; Maamar and Dubnau, 2005). Whether a specific cell becomes competent or not depends on the level of the master regulator ComK (van Sinderen et al., 1995), whose cellular amount is tightly controlled by a complex network of regulators acting on the transcriptional, post-transcriptional as well as on post-translational levels [for a detailed overview see Maier (2020)].

Transcription of the *comK* gene is controlled by three repressor proteins, Rok, CodY, and AbrB (Serror and Sonenshein, 1996; Hoa et al., 2002; Hamoen et al., 2003), moreover, *comK* transcription is activated by the transcriptional regulator DegU (Hamoen et al., 2000). Another important player for regulation of *comK* expression is Spo0A-P, which controls the levels of the AbrB repressor and additionally supports activation of *comK* expression by antagonizing Rok (Hahn et al., 1995; Mirouze et al., 2012). The presence of phosphorylated Spo0A directly links competence to other lifestyles, since Spo0A-P is also involved in pathways leading to sporulation or biofilm formation (Aguilar et al., 2010). When ComK levels reach a certain threshold, it binds its own promoter region to further increase its own expression, thereby creating a positive feedback loop, which leads to full activation of competence (Maamar and Dubnau, 2005; Smits et al., 2005).

ComK levels are also controlled post-transcriptionally by the Kre protein, which destabilizes the *comK* mRNA (Gamba et al., 2015). Post-translational regulation is achieved through the adapter protein MecA, which sequesters ComK and directs it toward degradation by the ClpCP protease (Turgay et al., 1998). During competence, this degradation is prevented by a small protein, ComS, which is expressed in response to quorum sensing (Nakano et al., 1991).

ComK activates expression of more than 100 genes (Berka et al., 2002; Hamoen et al., 2002; Ogura et al., 2002; Boonstra et al., 2020). Whereas a clear role in competence development has been assigned to many of the ComK regulon members, the roles of some ComK-dependent genes remain unclear. Similarly, many single deletion mutant strains were identified as competence deficient, for which the reasons for this deficiency are known. However, there are still many single deletion mutants, in which the reason for the loss of competence remains unknown. Typical examples for this are mutants lacking various RNases, namely RNase Y, RNase J, or PNPase (Luttinger et al., 1996; Figaro et al., 2013). Recently, a library of *B. subtilis* single gene deletion mutants was screened for various phenotypes, including competence development (Koo et al., 2017). This screen revealed 21 mutants with completely abolished competence. Out of those, 16 are known to be involved in the control of the ComK master regulator, DNA uptake or genetic recombination. However, in case of the other five competence-deficient strains the logical link to competence remains elusive.

Here, we have focused on some of these factors to investigate their role in genetic competence in more detail. We took advantage of the fact that artificial overexpression of ComK and ComS significantly increases transformation efficiency independently of traditional ComK and ComS regulation (Rahmer et al., 2015). This enables the identification of genes that are involved in competence development due to a function in *comK* expression or for other specific reasons downstream of ComK activity. We identified the YtrBCDEF ABC transporter, which is encoded in the *ytrGABCDEF* operon as an important player for *B. subtilis* differentiation, since its overexpression does not only result in a complete loss of competence by a so far unknown mechanism, it also affects the proper development of other lifestyles of *B. subtilis*. We hypothesize that the production of a thicker cell wall upon overexpression of the proteins encoded by the *ytrGABCDEF* operon is likely the cause of the observed competence and biofilm defects.

## Materials and Methods

### Bacterial Strains and Growth Conditions

*Bacillus subtilis* strains used in this study are listed in Table 1. Lysogeny broth (LB) (Sambrook et al., 1989) was used to grow *Escherichia coli* and *B. subtilis*, unless otherwise stated. When required, media were supplemented with antibiotics at the following concentrations: ampicillin 100 μg ml^–1^ (for *E. coli*) and chloramphenicol 5 μg ml^–1^, kanamycin 10 μg ml^–1^, spectinomycin 250 μg ml^–1^, tetracycline 12.5 μg ml^–1^, and erythromycin 2 μg ml^–1^ plus lincomycin 25 μg ml^–1^ (for *B. subtilis*). For agar plates, 15 g l^–1^ Bacto agar (Difco) was added.

**TABLE 1:** *Bacillus subtilis* strains used in this study.

**Strain**	**Genotype**	**Source^a^**
168	*trpC2*	Laboratory collection
BKE30420	*trpC2* Δ*ytrE::ermC*	Koo et al., 2017
BKE30430	*trpC2* Δ*ytrD::ermC*	Koo et al., 2017
BKE30440	*trpC2* Δ*ytrC::ermC*	Koo et al., 2017
BKE30450	*trpC2* Δ*ytrB::ermC*	Koo et al., 2017
PG389	*amyE::P_comG_-lacZ-gfp-cat*	Gamba et al., 2015
PG10^b^	*yvcA::*(*P_mtlA_-comKS*)	Reuß et al., 2017
DK1042	*comI*^Q12L^	Konkol et al., 2013
CCB434	Δ*rnjA::spc*	Figaro et al., 2013
CCB441	Δ*rny::spc*	Figaro et al., 2013
GP811	*trpC2* Δ*gudB::cat rocG::Tn10 spc amyE::(gltA-lacZ aphA3)* Δ*ansR::tet*	Flórez et al., 2011
GP1152	*trpC2* Δ*ansR::tetR*	GP811 → 168
GP1748	*trpC2* Δ*pnpA::aphA3*	Cascante-Estepa et al., 2016
GP2155	*trpC2* Δ*nrnA::aphA3*	See section “Materials and Methods”
GP2501	*trpC2* Δ*rny::spc*	CCB441 → 168
GP2506	*trpC2* Δ*rnjA::spc*	CCB434 → 168
GP2559	*comI*^Q12L^ Δ*ymdB::cat*	Kampf et al., 2018
GP2612	*trpC2* Δ*greA::aphA3*	See section “Materials and Methods”
GP2618	*trpC2 yvcA-P_mtlA_-comKS-ermC-hisI*	See section “Materials and Methods”
GP2620	*trpC2 yvcA-P_mtlA_-comKS-cat-hisI*	See section “Materials and Methods”
GP2621	*trpC2 yvcA-P_mtlA_-comKS-ermC-hisI* Δ*pnpA::aphA3*	GP1748 → GP2618
GP2624	*trpC2 yvcA-P_mtlA_-comKS-ermC-hisI* Δ*rny::spc*	GP2501 → GP2618
GP2626	*trpC2 yvcA-P_mtlA_-comKS-ermC-hisI* Δ*rnjA::spc*	GP2506 → GP2618
GP2630	*trpC amyE::P_comG_-lacZ-gfp-cat*	PG389 → 168
GP2640	*trpC2* Δ*ftsH::aphA3*	See section “Materials and Methods”
GP2641	*trpC2* Δ*ytrA::spc*	See section “Materials and Methods”
GP2643	*trpC2* Δ*comEC::spc*	See section “Materials and Methods”
GP2644	*trpC2* Δ*degU::aphA3*	See section “Materials and Methods”
GP2646	*trpC2* Δ*ytrGABCDEF::ermC*	See section “Materials and Methods”
GP2647	*trpC2* Δ*ytrA::ermC*	See section “Materials and Methods”
GP2652	*trpC2 yvcA-P_mtlA_-comKS-cat-hisI* Δ*ftsH::aphA3*	GP2640 → GP2620
GP2653	*trpC2 yvcA-P_mtlA_-comKS-cat-hisI* Δ*nrnA::aphA3*	GP2155 → GP2620
GP2654	*trpC2 yvcA-P_mtlA_-comKS-cat-hisI* Δ*greA::aphA3*	GP2612 → GP2620
GP2655	*trpC2 yvcA-P_mtlA_-comKS-cat-hisI* Δ*ytrA::spc*	GP2641 → GP2620
GP2659	*trpC2 yvcA-P_mtlA_-comKS-cat-hisI* Δ*comEC::spc*	GP2643 → GP2620
GP2660	*trpC2 yvcA-P_mtlA_-comKS-cat-hisI* Δ*degU::aphA3*	GP2644 → GP2620
GP2664	*trpC2 amyE::P_comG_-lacZ-gfp* Δ*ftsH::aphA3*	GP2640 → GP2630
GP2665	*trpC2 amyE::P_comG_-lacZ-gfp* Δ*nrnA::aphA3*	GP2155 → GP2630
GP2666	*trpC2 amyE::P_comG_-lacZ-gfp* Δ*greA::aphA3*	GP2612 → GP2630
GP2667	*trpC2 amyE::P_comG_-lacZ-gfp* Δ*ytrA::spc*	GP2641 → GP2630
GP2671	*trpC2 amyE::P_comG_-lacZ-gfp* Δ*comEC::spc*	GP2643 → GP2630
GP2672	*trpC2 amyE::P_comG_-lacZ-gfp* Δ*degU::aphA3*	GP2644 → GP2630
GP2700	*trpC2* Δ*ytrF::cat*	See section “Materials and Methods”
GP3186	*trpC2* Δ*ytrGABCDE::ermC*	See section “Materials and Methods”
GP3187	*trpC2* Δ*ytrF::cat* Δ*ytrA::ermC*	GP2647 → GP2700
GP3188	*trpC2* Δ*ytrB*	pDR244 → BKE30450
GP3189	*trpC2* Δ*ytrC*	pDR244 → BKE30440
GP3190	*trpC2* Δ*ytrD*	pDR244 → BKE30430
GP3191	*trpC2* Δ*ytrE*	pDR244 → BKE30420
GP3193	*trpC2* Δ*ytrA::ermC* Δ*ytrB*	See section “Materials and Methods”
GP3194	*trpC2* Δ*ytrA::ermC* Δ*ytrC*	See section “Materials and Methods”
GP3195	*trpC2* Δ*ytrA::ermC* Δ*ytrD*	GP2647 → GP3190
GP3196	*trpC2* Δ*ytrA::ermC* Δ*ytrE*	See section “Materials and Methods”
GP3197	*trpC2 ganA::P_xylA_-ytrF-aphA3*	pGP2184 → 168
GP3200	*trpC2 amyE::P_comG_-lacZ-gfp-cat ytrGABCDEF::ermC*	GP2646 → GP2630
GP3205	*trpC2* Δ*ytrCD::cat*	See section “Materials and Methods”
GP3206	*trpC2* Δ*ytrA::ermC* Δ*ytrB* Δ*ytrE*	See section “Materials and Methods”
GP3207	*comI*^Q12L^ Δ*ytrGABCDEF::ermC*	GP2646 → DK1042
GP3212	*comI*^Q12L^ Δ*ytrA::spc*	GP2641 → DK1042
BLMS2	*trpC2* Δ*ytrCD*	See section “Materials and Methods”
BLMS3	*trpC2* Δ*ytrA::erm* Δ*ytrCD*	See section “Materials and Methods”

*^*a*^Arrows indicate construction by transformation. ^*b*^This genome-reduced strain [see Reuß et al. (2017) for details] was used to amplify the *P_*mtlA*_-comKS* cassette.*

### DNA Manipulation and Strain Construction

S7 Fusion DNA polymerase (Mobidiag, Espoo, Finland) was used as recommended by the manufacturer. DNA fragments were purified using the QIAquick PCR Purification Kit (Qiagen, Hilden, Germany). DNA sequences were determined by the dideoxy chain termination method (Sambrook et al., 1989). Chromosomal DNA from *B. subtilis* was isolated using the peqGOLD Bacterial DNA Kit (Peqlab, Erlangen, Germany) and plasmids were purified from *E. coli* using the NucleoSpin Plasmid Kit (Macherey-Nagel, Düren, Germany). Oligonucleotides used in this study are listed in Supplementary Table 1. Deletion of the *degU*, *comEC*, *ftsH*, *greA*, *ytrA*, *nrnA*, and *ytrF* genes as well as *ytrG-ytrE*, and *ytrGABCDEF* regions was achieved by transformation with PCR products containing an antibiotic resistance cassette flanked by up- and downstream fragments of the target genes as described previously (Youngman, 1990; Guérout-Fleury et al., 1995; Wach, 1996). The identity of the modified genomic regions was verified by DNA sequencing. To construct strains GP2618 and GP2620 harboring the P*_mtlA_*-*comKS* cassette coupled to the antibiotic resistance gene, we first amplified P*_mtlA_*-*comKS* from strain PG10 (Reuß et al., 2017) as well as the resistance genes from pDG646 and pGEM-cat, respectively (Youngman, 1990; Guérout-Fleury et al., 1995) and the genes flanking the intended integration site, i.e., *yvcA* and *hisI* from *B. subtilis* 168. Subsequently, those DNA fragments were fused in another PCR reaction and the final product was used to transform *B. subtilis* 168. Correct insertion was verified by PCR amplification and sequencing. Markerless deletions of *ytrB*, *ytrC*, *ytrD*, and *ytrE* genes were generated using the plasmid pDR244 as previously described (Koo et al., 2017). In short, strains BKE30450, BKE30440, BKE30430, and BKE30420 were transformed with plasmid pDR244 and transformants were selected on LB agar plates supplemented with spectinomycin at 30°C. Transformants were then streaked on plain LB agar plates and incubated at 42°C to cure the plasmid, which contains a thermo-sensitive origin of replication. Single colonies were screened for spectinomycin and erythromycin/lincomycin sensitivity. The markerless deletion of *ytrCD* was achieved using the cre-lox system. First, Lox71 and Lox66 sites were attached to the kanamycin resistance gene using primers CZ168/169. The resulting PCR product was cut with *Eco*RI and *Xba*I and ligated with plasmid pBluescript II that had been cut with the same enzymes, yielding plasmid pGP2514. The kanamycin resistance cassette flanked by Lox71 and Lox66 sites was subsequently amplified from pGP2514 using primers CZ200/201. Next, 800 bp up- and downstream of *ytrCD* were amplified using primers MB198/LMS262 and MB201/LMS260, respectively. Primers LMS262 and LMS260 contained overhangs complementary to primers CZ200/201. The three fragments were subsequently fused using primers MB198/MB201, the resulting PCR product transformed into the *B. subtilis* wildtype strain 168 and transformants selected on SP medium containing kanamycin. The strain was cured from the kanamycin resistance cassette using plasmid pDR244 as described above, resulting in the construction of strain BLMS2. Markerless deletion was confirmed by PCR with primers flanking the deletion site. The resulting strains GP3188, GP3189, GP3190, GP3191, and BLMS2 were used for subsequent deletion of the *ytrA* gene. For the construction of GP3193, GP3194, and GP3196, the *ytrA* deletion cassette was amplified using primers MB66/69 and genomic DNA of strains GP3188, GP3189, and GP3191, respectively, as template. For the construction of BLMS3, the Δ*ytrA:erm* region was amplified using primers MB173/70 and genomic DNA of strain GP2647. The corresponding *ytrA* deletion cassettes were subsequently transformed into GP3188, GP3189, GP3191, and BLMS2. Strain GP3195 was constructed by transformation with genomic DNA of the *ytrA* deletion strain. Deletion of the *ytrA* gene and preservation of selected markerless deletions were confirmed *via* PCR. To construct GP3206, the Δ*ytrA:erm* Δ*ytrB* region of strain GP3193 was amplified using primers MB66 and MB180 and transformed into GP3191.

### Transformation of *Bacillus subtilis* Strains

Transformation experiments were conducted based on the two-step protocol as described previously (Kunst and Rapoport, 1995). Briefly, cells were grown at 37°C at 200 rpm in 10 ml MNGE medium containing 2% glucose, 0.2% potassium glutamate, 100 mM potassium phosphate buffer (pH 7), 3.4 mM trisodiumcitrate, 3 mM MgSO_4_, 42 μM ferric ammonium citrate, 0.24 mM L-tryptophan and 0.1% casein hydrolyzate. During the transition from exponential to stationary phase, the culture was diluted with another 10 ml of MNGE medium (without casein hydrolyzate) and incubated for 1 h at 37°C with shaking. For transformation experiments with strain GP3197, 0.5% xylose was added to both media. Afterward, 250 ng of chromosomal DNA was added to 400 μl of cells and incubated for 30 min at 37°C. One hundred microliter of Expression mix (2.5% yeast extract, 2.5% casein hydrolyzate, 1.22 mM tryptophan) was added and cells were grown for 1 h at 37°C, before spreading onto selective LB plates containing appropriate antibiotics.

Transformation of strains harboring P*_mtlA_-comKS*, in which the expression of *comK* and *comS* is induced in the presence of mannitol, was performed as previously described (Rahmer et al., 2015). Briefly, an overnight culture was diluted in 5 ml LB to an initial OD_600_ of 0.1 and incubated at 37°C and 200 rpm. After 90 min of incubation, 5 ml of fresh LB containing 1% mannitol and 5 mM MgCl_2_ were added and the bacterial culture was incubated for an additional 90 min. Cells were then pelleted by centrifugation for 10 min at 2,000 × *g* and the pellet was re-suspended in the same amount of fresh LB medium. 1 ml aliquots were distributed into 1.5 ml reaction tubes and 250 ng of chromosomal DNA was added to each of them. The cell suspension was incubated for 1 h at 37°C and transformants were selected on LB plates as described above.

### Plasmid Construction

All plasmids used in this study are listed in Table 2. *E. coli* DH5a (Sambrook et al., 1989) was used for plasmid constructions and transformation using standard techniques (Sambrook et al., 1989). To overproduce the YtrF protein, the *ytrF* gene was placed under the control of a xylose inducible promotor. For this purpose, we cloned the *ytrF* gene into the backbone of pGP888 *via* the *Xba*I and *Kpn*I sites (Diethmaier et al., 2011).

**TABLE 2:** Plasmids used in this study.

**Plasmid**	**Relevant characteristics**	**Primers**	**References**
pDR244	*cre* + Ts origin	–	Koo et al., 2017
pGEM-cat	Amplification of the *cat* cassette	–	Youngman, 1990
pDG646	Amplification of the *ermC* cassette	–	Guérout-Fleury et al., 1995
pDG780	Amplification of the *aphA3* cassette	–	Guérout-Fleury et al., 1995
pDG1726	Amplification of the *spc* cassette	–	Guérout-Fleury et al., 1995
pGP888	*ganA:*P*_xylA_*; *aphA3*	–	Diethmaier et al., 2011
pGP2184	pGP888-*ytrF*	MB186/MB187	This study
pGP2514	Amplification of *aphA3*-lox for the cre-lox system	CZ200/CZ201	This study

### Quantitative Real-Time PCR

For the isolation of RNA, a single colony was used to inoculate 4 ml LB medium containing the appropriate antibiotics. The cells were grown over the day at 37°C with agitation and used to inoculate 10 ml MNGE defined medium and incubated overnight. The next day, 100 ml MNGE medium were inoculated to an OD_600_ of 0.1 and the cells were incubated at 37°C until they reached an OD_600_ of 1.0. 25 ml of each culture were mixed with 15 ml frozen killing buffer (20 mM Tris, pH 7.5, 5 mM MgCl_2_, 20 mM NaN_3_), followed by a 5-min centrifugation step at 8,000 rpm and 4°C. Pellets were snap-frozen in liquid nitrogen and stored at −80°C. Disruption of cells and isolation of RNA was carried out as previously described (Meinken et al., 2003). 5 μg isolated RNA were digested with 5 μl DNase I (1 U/μl, Thermo Scientific) for 40 min at 37°C. The reaction was stopped by adding 2.5 μl 25 mM EDTA and incubating the samples at 65°C for 10 min. To verify that the isolated RNA is free of DNA, a check PCR was performed using primers KG44/KG45. Genomic DNA from *B. subtilis* 168 was used as control.

Quantitative real-time PCR (qRT-PCR) was carried out using the One-Step reverse transcription PCR kit, the Bio-Rad iCycler and the Bio-Rad iQ5 software (Bio-Rad, Munich, Germany). Three technical and three biological repeats were performed. Primers KG44/45 and KG42/43 were used to determine transcript amounts of the ribosomal genes *rpsE* and *rpsJ*, respectively, which were used as internal controls. Transcript amounts for *ytrE* and *ytrF* were monitored using primers MB219/220 and MB224/225, respectively. The average of the cycle threshold (C_T_) values of *rpsE* and *rpsJ* were used to normalize the C_T_-values obtained for *ytrE* and *ytrF*. For each strain, the fold changes of *ytrE* and *ytrF* expression were calculated using the ΔΔC_T_-method.

### Biofilm Assay

To analyze biofilm formation, selected strains were grown in LB medium to an OD_600_ of about 0.5–0.8 and 10 μl of the culture were spotted onto MSgg agar plates (Branda et al., 2001). Plates were incubated for 3 days at 30°C.

### Fluorescence Microscopy

For fluorescence microscopy imaging, *B. subtilis* cultures were grown in 10 ml MNGE medium until the transition from exponential to stationary phase and then diluted with another 10 ml of MNGE medium as described for the transformation experiments (see section “Transformation of *Bacillus subtilis* Strains”). 2 ml of the bacterial culture were harvested and resuspended in 500 μl PBS. The cell suspension was mixed with 25 μl of 100 μg/ml nile red solution to stain the bacterial membranes. 5 μl of cells were pipetted on microscope slides coated with a thin layer of 1% agarose and covered with a cover glass. Fluorescence images were obtained with the AxioImager M2 fluorescence microscope, equipped with the digital camera AxioCam MRm and an EC Plan-NEOFLUAR 100X/1.3 objective (Carl Zeiss, Göttingen, Germany). Filter sets 38 (EX BP 470/40, FT 495, EM BP 525/50; Carl Zeiss) and 43 (EX BP 545/25, FT 579, EM BP 605/70; Carl Zeiss) were applied for GFP and nile red detection, respectively. Images were processed with the AxioVision Rel 4.8 software. Ratio of GFP expressing cells to the total number of cells was determined by manual examination from at least six independent growth experiments. For each experiment, the ratio of GFP expressing wildtype cells was set to one and used to calculate the relative GFP fluorescence for each mutant of the same experiment.

### Transmission Electron Microscopy

To examine cell wall thickness of *B. subtilis* strains, cells were prepared for Transmission Electron Microscopy (TEM) as previously described (Rismondo et al., 2021). An overnight culture was inoculated to an OD_600_ of 0.05 in 30 ml MNGE medium and grown to an OD_600_ of 0.6 ± 0.1 at 37°C and 200 rpm. Cells were centrifuged for 10 min at 4,000 rpm to obtain a 100 μl cell pellet, which was then washed twice in phosphate-buffered saline (PBS, 127 mM NaCl, 2.7 mM KCl, 10 mM Na_2_HPO_4_, 1.8 mM KH_2_PO_4_, pH 7.4) and fixed overnight in 2.5% (w/v) glutaraldehyde at 4°C. Cells were mixed with 1.5% (w/v, final concentration in PBS) molten Bacto-Agar, kept liquid at 55°C. After solidification, the resulting agar block was cut into 1 mm^3^ pieces. A dehydration series was performed (15% aqueous ethanol solution for 15 min, 30, 50, 70, and 95% for 30 min and 100% for 2 × 30 min) at 0°C, followed by an incubation step in 66% LR white resin mixture (v/v, in ethanol) (Plano, Wetzlar, Germany) for 2 h at room temperature and embedment in 100% LR-White solution overnight at 4°C. One agar piece was transferred to a gelatin capsule filled with fresh LR-white resin, which was subsequently polymerized at 55°C for 24 h. The gelatin capsule was shaped into a truncated pyramid using a milling tool (TM 60, Fa. Reichert and Jung, Vienna, Austria) An ultramicrotome (Reichert Ultracut E, Leica Microsystems, Wetzlar, Germany) and a diamond knife (Delaware Diamond Knives, Wilmington, DE, United States) were subsequently used to obtain ultrathin sections (80 nm) of the samples. The resulting sections were mounted onto mesh specimen grids (Plano, Wetzlar, Germany) and stained with 4% (w/v) uranyl acetate solution (pH 7.0) for 10 min. Microscopy images were taken on a Jeol JEM 1011 transmission electron microscope (Jeol Germany GmbH, Munich, Germany) at 80 kV, with a magnification of 30,000 and recorded with an Orius SC1000 CCD camera (Gatan Inc., Pleasanton, CA, United States). For each replicate, 20 cells were photographed and cell wall thickness was measured at three different locations using the software ImageJ (Rueden et al., 2017).

## Results

### ComK-Dependent and –Independent Functions of Proteins Are Required for the Development of Genetic Competence

Genetic work with *B. subtilis* is facilitated by the development of genetic competence, a process that depends on a large number of factors. While the specific contribution of many proteins to the development of competence is well understood, this requirement has not been studied for many other factors. In particular, several RNases (RNase Y, RNase J1, and PNPase) are required for competence, and the corresponding mutants have lost the ability to become naturally competent (Luttinger et al., 1996; Figaro et al., 2013). We are interested in the reasons for the loss of competence in these mutant strains, as well as in other single gene deletion mutants, in which the impairment in the development of natural competence is not understood (Koo et al., 2017). Therefore, we first tested the roles of the aforementioned RNases (encoded by the *rny*, *rnjA*, *pnpA, and nrnA* genes) as well as of the transcription elongation factor GreA, the metalloprotease FtsH and the transcription factor YtrA (Koo et al., 2017) for the development of genetic competence. For this purpose, we compared the transformation efficiencies of the corresponding mutant strains to that of a wild type strain. The *comEC* and *degU* mutants, which have completely lost their genetic competence for different reasons, were used as controls. The ComEC protein is responsible for the transport of the DNA molecule across the cytoplasmic membrane. Loss of ComEC blocks competence, but it should not affect the global regulation of competence development and expression of other competence factors (Draskovic and Dubnau, 2005). In contrast, DegU is a transcription factor required for the expression of the key regulator of competence, ComK, and thus indirectly also for the expression of all other competence genes (Hamoen et al., 2000; Shimane and Ogura, 2004). Our analysis confirmed the significant decrease in transformation efficiency for all tested strains (see Table 3). For five out of the seven strains, as well as the two control strains, competence was abolished completely, whereas transformation of strains GP2155 (Δ*nrnA*) and GP1748 (Δ*pnpA*) was possible, but severely impaired as compared to the wild type strain. These results confirm the implication of these genes in the development of genetic competence.

**TABLE 3:** Effect of gene deletions on the development of genetic competence in dependence of the competence transcription factor ComK^a^.

	**Wild type**	***P_mtlA_-comKS***
	
**Mutant**	**Colonies per μg of DNA**	
Wild type	138,60017,006	47,9528,854
Δ*degU*	00	60,85313,693
Δ*comEC*	00	00
Δ*nrnA*	1,689316	34,9336,378
Δ*ftsH*	00	00
Δ*greA*	00	00
Δ*rny*	00	00
Δ*rnjA*	00	00
Δ*pnpA*	176	29319
Δ*ytrA*	00	467278

*^*a*^Cells were transformed with chromosomal DNA of strain GP1152 harboring a tetracycline resistance marker as described in section “Materials and Methods.”*

The proteins that are required for genetic competence might play a more general role in the control of expression of the competence regulon (as known for the regulators that govern *comK* expression and ComK stability, e.g., the control protein DegU), or they may have a more specific role in competence development such as the control protein ComEC. To distinguish between these possibilities, we introduced the mutations into a strain that allows for the inducible overexpression of the *comK* and *comS* genes. The overexpression of *comK* and *comS* allows transformation in rich medium and hence facilitates the transformation of some competence mutants (Rahmer et al., 2015). For this purpose, we first constructed strains that contain mannitol inducible *comK* and *comS* genes fused to resistance cassettes (GP2618 and GP2620, for details see section “Materials and Methods”). Subsequently, we deleted our target genes in this genetic background and assayed transformation efficiency after induction of *comKS* expression (for details see section “Materials and Methods”). In the *B. subtilis* wild type strain 168, deletion of *degU* leads to a complete loss of competence, while the transformation efficiency of a *degU* mutant overexpressing *comKS* is comparable to its isogenic wild type strain. This suggests that DegU affects competence only by its role in *comK* expression and that DegU is no longer required in the strain with inducible *comKS* expression. In contrast, the competence of the *comEC* mutant could not be restored by the overexpression of *comKS*, reflecting the role of the ComEC protein on a process downstream of ComK, namely on DNA uptake (see Table 3). Of the tested strains, only the *nrnA* mutant showed a transformation efficiency similar to that of the isogenic control strain with inducible *comKS* expression. This observation suggests that nanoRNase A might be involved in the control of *comK* expression. In contrast, the *ftsH, greA, rny*, and *rnjA* mutants did not show any transformants even upon *comKS* overexpression, indicating that the corresponding proteins act downstream of ComK. Finally, we have observed a small but reproducible improvement of competence for the *pnpA* and *ytrA* mutants, indicating that PNPase and YtrA might affect *comK* expression. This finding is particularly striking in the case of the *ytrA* mutant, since this strain did not yield a single transformant in the 168 wild type background (see Table 3). However, the low number of transformants obtained with *pnpA* and *ytrA* mutants as compared to the isogenic wild type strain suggests that PNPase and the YtrA transcription factor play also a role downstream of ComK.

**FIGURE 1:**
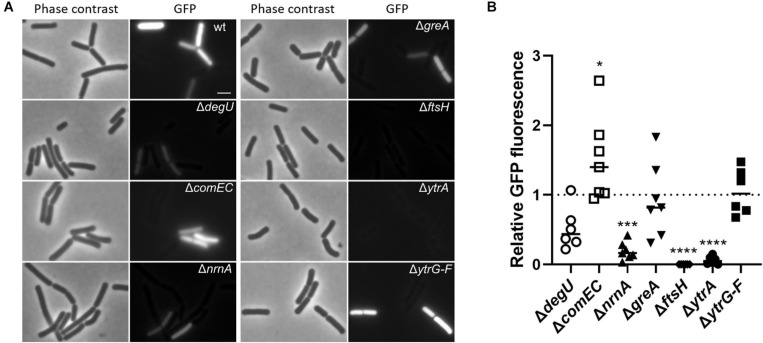
Effect of gene deletions on the expression of a P*_comG_-gfp* translational fusion as a readout for the activity of the competence transcription factor ComK. **(A)** Strains harboring the P*_comG_-gfp* construct were grown in competence inducing medium as described in section “Materials and Methods.” Cells were analyzed by phase contrast and fluorescence microscopy and representative images are shown. Scale bar is 2 μm. **(B)** The percentage of GFP-expressing cells was determined for each strain. The ratio of GFP-expressing cells of each mutant compared to the wildtype strain was calculated and the result of at least six independent experiments plotted. For statistical analysis, a one-way ANOVA followed by a Dunnett’s multiple comparison test was used (*p ≤ 0.05, ***p ≤ 0.001, and ****p ≤ 0.0001).

ComK activates transcription of many competence genes including *comG* (van Sinderen et al., 1995). Therefore, as a complementary approach to verify the results shown above, we decided to assess ComK activity using a fusion of the *comG* promoter to a promoterless GFP reporter gene (Gamba et al., 2015). For this purpose, we deleted the selected genes in strain GP2630 containing the P*_comG_*-*gfp* construct. We grew the cells in competence inducing medium using the two-step protocol as we did in the previous transformation experiment. At the time point when DNA would be added to the cells during the transformation procedure, we assessed *comG* promoter activity in the cells using fluorescence microscopy. Since expression of ComK and thus also activation of competence takes place only in a subpopulation of cells (Smits et al., 2005), we determined the ratio of *gfp* expressing cells for each mutant strain compared to the wildtype strain as an indication of ComK activity (see Figure 1). Since RNase mutants tend to form chains, thus making it difficult to study fluorescence in individual cells, we did not include the RNase mutants for this analysis.

In the wild type strain GP2630, about 40% of the cells expressed GFP, similar to previously published results (She et al., 2020), and slightly higher numbers were obtained for the control strain lacking ComEC, which is not impaired in *comK* and subsequent *comG* expression. In contrast, the control strain lacking DegU showed decreased amount of GFP expressing cells as compared to the wild type (Figure 1), which reflects the role of DegU on the activation of *comK* expression. In agreement with our previous finding that nanoRNase A affects *comK* expression or ComK activity, only about 4% of *nrnA* mutant cells showed expression from P*_comG_*-*gfp*. Thus, the nanoRNase A encoded by *nrnA* seems to play a role in the regulation of *comK* expression, which has not been described so far. For the strain lacking GreA, we observed similar rates of GFP expressing cells as in the wild-type strain, indicating that ComK activation is not the problem, which causes loss of competence in the *greA* mutant. Surprisingly, we did not find any single cell expressing GFP for the *ftsH* mutant, where ComK expression does not seem to be the cause of competence deficiency as indicated by the previous transformation experiment. Additionally, we observed significantly decreased numbers of GFP producing cells for the *ytrA* deletion mutant, in which the competence deficiency could only be slightly restored by *comKS* overexpression. This observation suggests that FtsH and YtrA could potentially play a dual role in the development of genetic competence. On one hand, they both seem to be required for ComK activity but on the other hand, they also seem to have a ComK-independent function. However, it is also possible that the overexpression of *comK* and *comS* using the P*_mtlA_* promoter does not lead to the production of sufficient amounts of ComK to fully restore competence of the *ftsH* and *ytrA* mutants. The *ytrA* gene encodes a transcription factor, whose physiological function is poorly understood (Salzberg et al., 2011). Therefore, we focused our further work on understanding the role of YtrA on the development of genetic competence.

### Overexpression of the YtrBCDEF ABC Transporter Inhibits Genetic Competence

The *ytrA* gene encodes a negative transcription regulator of the GntR family, which binds to the inverted repeat sequence AGTGTA-13bp-TACACT (Salzberg et al., 2011). In the *B. subtilis* genome, this sequence is present in front of two operons, its own operon *ytrGABCDEF* and *ywoBCD*. The deletion of *ytrA* leads to an overexpression of these two operons (Salzberg et al., 2011). It is tempting to speculate that overexpression of one of these operons is the cause for the loss of competence in the *ytrA* mutant. In case of the *ytrA* mutant used in this study, the expression of the downstream genes, namely *ytrBCDEF*, is controlled by the promoter of the *ermC* gene, which was inserted into the *ytrA* locus. This results in a 335- and 566-fold higher expression of *ytrE* and *ytrF*, respectively, in the *ytrA* mutant as compared to the *B. subtilis* wildtype strain (Supplementary Figure 1). To test whether the overexpression of *ytrBCDEF* leads to the competence deficiency of the *ytrA* mutant, we constructed strain GP2646, where the complete *ytrGABCDEF* operon is replaced by the *ermC* gene and assayed its genetic competence. This revealed that although deletion of *ytrA* fully blocks genetic competence, the transformation efficiency of the strain lacking the whole operon is comparable to the wild type strain 168 (Table 4). We conclude that overexpression of the *ytrGABCDEF* operon causes the loss of competence in the *ytrA* mutant strain. In addition, we assessed ComK activity in the mutant lacking the *ytrGABCDEF* operon, using the expression of the P*_comG_*-*gfp* fusion as a readout. As observed for the wild type, about 40% of the mutant cells expressed *comG*, indicating that ComK is fully active in the mutant (Figure 1), and that the reduced activity in the *ytrA* mutant results from the overexpression of the operon. Initially we also aimed to delete the *ywoBCD* operon to assess a potential involvement in genetic competence, however several attempts to construct such a strain failed. As we were already able to show that the overexpression of the *ytr* operon causes the loss of competence in the *ytrA* mutant, we decided not to continue with this second YtrA-controlled operon.

**TABLE 4:** Effect of gene deletions in the *ytrGABCDEF* operon on the development of genetic competence^a^.

**Mutant**	**Colonies per μg of DNA**
Wild type	138,60017,006
Δ*ytrGABCDEF*	114,73314,408
Δ*ytrA*	00
Δ*ytrAB*	00
Δ*ytrAC*	00
Δ*ytrAD*	242
Δ*ytrAE*	13751
Δ*ytrAF*	10,180549
P*_xyl_*-*ytrF*	137,53326,595
Δ*ytrGABCDE*	108,46714,836
Δ*ytrABE*	30988
Wild type	142,60039,074
Δ*ytrACD*	1.72.4

*^*a*^Cells were transformed with chromosomal DNA of strain GP1152 harboring a tetracycline resistance marker as described in section “Materials and Methods.”*

**FIGURE 2:**
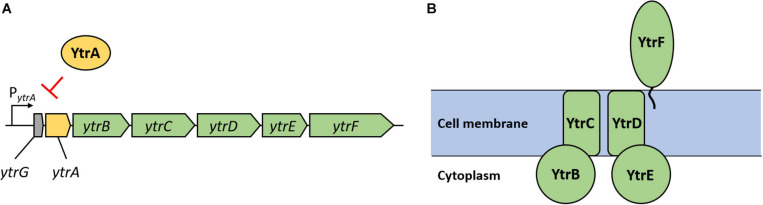
Genetic organization of the *ytrGABCDEF* operon and organization of the putative ABC transporter YtrBCDEF. **(A)** Reading frames are depicted as arrows with respective gene names. Green arrows indicate genes encoding proteins suggested to form the ABC transporter; the yellow arrow indicates the gene coding for the repressor YtrA and the gray arrow indicates the small open reading frame called *ytrG*. The map is based on information provided in Salzberg et al. (2011). **(B)** Organization of the putative ABC transporter YtrBCDEF as suggested by Yoshida et al. (2000). YtrB and YtrE are nucleotide binding proteins, YtrC and YtrD membrane spanning proteins and YtrF is a substrate binding protein. The role and localization of the YtrG peptide remain elusive.

The *ytr* operon consists of seven genes (see Figure 2A). Five proteins encoded by this operon (YtrB, YtrC, YtrD, YtrE, and YtrF) are components of a putative ABC transporter (see Figure 2B), which was suggested to play a role in acetoin utilization (Quentin et al., 1999; Yoshida et al., 2000). YtrB and YtrE are supposed to be nucleotide binding proteins, YtrC and YtrD membrane spanning proteins and YtrF the substrate binding protein. Finally, *ytrG* is another open reading frame, which is located upstream of *ytrA* and encodes a peptide of 45 amino acids, which is probably not part of the ABC transporter (Salzberg et al., 2011). The expression of the *ytr* operon is usually kept low due to transcriptional repression by YtrA. This repression is naturally relieved in response to several lipid II-binding antibiotics or during cold shock (Beckering et al., 2002; Salzberg et al., 2011; Wenzel et al., 2012).

To test the involvement of the individual components of the putative YtrBCDEF ABC transporter in the development of genetic competence, we constructed double mutants of *ytrA* together with each of the other genes of the operon, i.e., *ytrB, ytrC, ytrD, ytrE*, and *ytrF*, and assessed their genetic competence. The results revealed that most of the double mutants are deficient in genetic transformation, as observed for the single *ytrA* mutant GP2647 (Table 4). However, strain GP3187 with deletions of *ytrA* and *ytrF* but still overexpressing all the other parts of the transporter had partially restored competence. YtrF could thus be a major player for the loss of competence in the overexpressing strain.

To further test the role of YtrF overexpression for the loss of competence, we used two different approaches. First, we constructed a strain with artificial overexpression of *ytrF* from a xylose inducible promoter (GP3197) and second, we created a strain with deletion of all other components (*ytrGABCDE*) of the operon, leaving only overexpressed *ytrF* (GP3186). Overexpression of *ytrF* in strain GP3186 was confirmed by qRT-PCR analysis (Figure 2). In contrast to our expectations, competence was not blocked in any of the two strains, suggesting that increased levels of YtrF alone are not enough to block the competence and that YtrF might need assistance from other components of the putative transporter for its full action and/or proper localization. The *ytr* operon encodes two putative nucleotide binding proteins (YtrB and YtrE) and two putative membrane spanning proteins (YtrC and YtrD), whereas YtrF is the only substrate binding protein that interacts with the transmembrane proteins. Therefore, we hypothesized that YtrF overexpression might only block genetic competence if the protein is properly localized in the membrane *via* YtrC and YtrD. To check this possibility, we constructed strains GP3206 and BLMS3 lacking YtrA and the nucleotide binding proteins YtrB and YtrE or the membrane proteins YtrC and YtrD, respectively, and tested their transformability. qRT-PCR analysis was again used to confirm that *ytrF* is overexpressed in strains GP3206 and BLMS3 as compared to the wildtype strain 168 (Supplementary Figure 1). Strain GP3206 showed very few transformants, suggesting that the overexpression of *ytrC*, *ytrD*, and *ytrF*, encoding the remaining transporter components, are sufficient to cause the loss of competence. Surprisingly, similar results were obtained for a *B. subtilis* strain lacking YtrA, YtrC, and YtrD. We thus conclude that the observed loss of competence as a result of YtrF overexpression does not depend on the nucleotide binding proteins or the transmembrane proteins. Since we did not observe a loss of competence for *B. subtilis* strains, in which only YtrF is overexpressed, we hypothesize that YtrF might require assistance of another, unknown factor.

### Overexpression of the *ytrGABCDEF* Operon Leads to Alterations in Colony Morphology During Biofilm Formation

**FIGURE 3:**
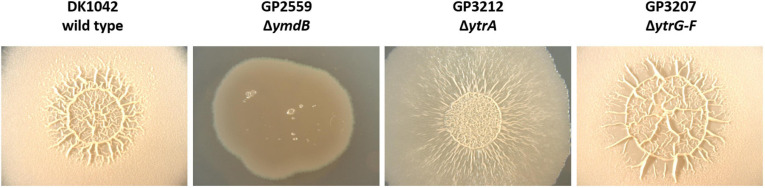
Biofilm formation is affected by *ytrA* deletion. Biofilm formation was examined in the wild type strain DK1042 and respective deletion mutants of *ymdB* (GP2559), *ytrA* (GP3212), and *ytrGABCDEF* (GP3207). The biofilm assay was performed on MSgg agar plates as described in section “Materials and Methods.” The plates were incubated for 3 days at 30°C. All images were taken at the same magnification.

*Bacillus subtilis* can employ various lifestyles which are tightly interconnected through regulatory proteins (Lopez et al., 2009). Therefore, we anticipated that the overexpression of the YtrBCDEF transporter might also affect other lifestyles of *B. subtilis*. Indeed, it was previously shown that the *ytrA* mutant has a reduced sporulation efficiency (Koo et al., 2017). We thus decided to examine the effect of *ytrA* deletion on biofilm formation. To that end, we deleted the *ytrA* gene or the whole *ytrGABCDEF* operon from the biofilm-forming strain DK1042 (Konkol et al., 2013). We then tested the biofilm formation of the resulting strains on biofilm inducing MSgg agar (Branda et al., 2001). As expected, the wild type strain DK1042 formed structured colonies that are indicative of biofilm formation. In contrast, the negative control GP2559, a *ymdB* mutant that is known to be defective in biofilm formation (Kampf et al., 2018), formed completely smooth colonies. The biofilm formed by the *ytrA* mutant GP3212 was less structured, more translucent and with only some tiny wrinkles on its surface, indicating that biofilm formation was slightly inhibited but not fully abolished upon loss of YtrA. In contrast, strain GP3207 lacking the complete *ytrGABCDEF* operon formed a biofilm indistinguishable from the one of the parental strain DK1042 (see Figure 3). This observation suggests that overexpression of components of the YtrBCDEF ABC transporter could interfere with biofilm formation in *B. subtilis*.

### Overexpression of the *ytr* Operon Increases Cell Wall Thickness

In previous experiments, we have shown that the overexpression of the *ytr* operon interferes with the development of genetic competence and, to some extent, with biofilm formation. However, it remains unclear why competence and biofilm formation are affected by the overexpression of the *ytr* operon. The *ytr* operon is repressed under standard laboratory conditions by the YtrA transcription regulator and this repression is naturally relieved upon exposure to very specific stress conditions such as cell wall targeting antibiotics and cold shock (Beckering et al., 2002; Cao et al., 2002; Mascher et al., 2003; Salzberg et al., 2011; Nicolas et al., 2012; Wenzel et al., 2012). The possible link between antibiotic resistance, genetic competence, and biofilm formation is not apparent, however, cell wall properties might provide an answer. Indeed, it has been shown that wall teichoic acids, the uppermost layer of the cell wall, are important for biofilm formation and for DNA binding during transformation (Bucher et al., 2015; Mirouze et al., 2018; Zhu et al., 2018).

**FIGURE 4:**
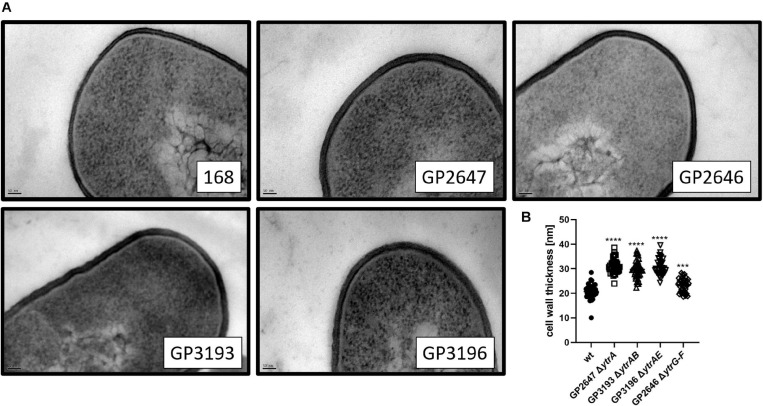
Cell wall thickness is increased in *ytrA*, *ytrAB*, and *ytrAE* mutants. **(A)** Shown are representative transmission electron microscopy images of the wild type strain 168, the *ytrA* mutant (GP2647), the *ytrAB* (GP3193), and the *ytrAE* (GP3196) double mutants and the *ytrGABCDEF* mutant (GP2646). Scale bar is 50 nm. **(B)** Cell wall thickness of *B. subtilis* wild type and *ytr* mutants. The cell wall thickness of 40 individual cells per strain was measured as described in section “Materials and Methods”and plotted. For statistical analysis, a one-way ANOVA followed by a Dunnett’s comparison test was used (***p ≤ 0.001 and ****p ≤ 0.0001).

To test the hypothesis that overexpression of the putative ABC transporter encoded by the *ytrGABCDEF* operon affects cell wall properties of *B. subtilis* cells, we compared the cell morphology of the wild type, the non-competent *ytrA*, *ytrAB*, *ytrAE* mutants as well as the competent *ytrGABCDEF* mutant lacking the complete operon by TEM. While the wild type strain showed an average cell wall thickness of 21 nm, which is in agreement with previous studies (Beveridge and Murray, 1979), the *ytrA* (GP2647) mutant showed a significant increase in cell wall thickness with an average of 31 nm. *B. subtilis* strains lacking one of the nucleotide binding proteins of the transporter, YtrB or YtrE, in addition to YtrA also produced a thicker cell wall with an average thickness of 30 nm and 31 nm, respectively. In contrast, such an increase was not observed for the whole operon mutant (GP2646) that had an average cell wall thickness of 23 nm (see Figure 4). These observations are in agreement with the hypothesis that the overexpression of the YtrBCDEF ABC transporter affects cell wall properties and could potentially explain the loss of genetic competence and the minor biofilm formation defect of the *ytrA* mutant.

## Discussion

Genetic competence is a multifactorial process in the Gram-positive organism *B. subtilis*. In a genome-wide study, several single deletion mutants were identified, which are significantly impaired in competence development (Koo et al., 2017). However, it remains to be determined why the deletion of the corresponding genes affect this process. Here, we aimed to investigate the role of the transcription elongation factor GreA, the metalloprotease FtsH, the transcriptional regulator YtrA and the RNases RNase Y, RNase J1, PNPase, and nanoRNase A on competence development in *B. subtilis*. In accordance with previous studies, deletion of *greA, ftsH, ytrA, rny*, and *rnjA* resulted in a complete loss of competence and the competence of strains lacking the PNPase or nanoRNase A was severely reduced (Figaro et al., 2013; Koo et al., 2017). The overexpression of *comKS*, coding for the master regulator of competence ComK and the small antiadapter protein ComS, could not restore the competence of the *rny*, *rnjA*, and *greA* mutants, suggesting that RNase Y, RNase J1, and GreA act downstream of the control of *comK* expression. In contrast, overexpression of *comKS* restored the competence efficiency of the *nrnA* mutant to a level comparable to the isogenic wild type strain and we hypothesize that nanoRNase A directly or indirectly affects *comK* expression. Lack of the metalloprotease FtsH led to a complete loss of genetic competence that could not be restored by the overexpression of *comKS* suggesting that FtsH acts downstream of ComK. However, we could not detect any expression from the *comG* promoter, which contradicts the first result. We hypothesize that FtsH might have a dual role: on one hand, it could influence the expression of *comK* and on the other hand, it could have a ComK-independent role. However, it is also possible that ComK is not properly expressed in the *ftsH* mutant or that FtsH is involved in the turnover of MecA, Rok or AbrB leading to a reduced expression of *comK* or an enhanced degradation of ComK. Further studies are required to understand the precise role of FtsH on competence development in *B. subtilis*. For the *pnpA* and *ytrA* mutants, we observed a slight improvement of competence, suggesting that PNPase and YtrA could play a role in *comK* expression control. However, the genetic competence could not be fully restored by *comKS* overexpression, indicating that ComK might not be the only factor contributing to the competence deficiency of the *pnpA* and *ytrA* mutants. Again, elevated ComK levels might not be sufficient to completely suppress the competence deficiency of these two mutants.

The transcriptional repressor YtrA is encoded in the *ytrGABCDEF* operon, whose expression is induced in the presence of cell wall-acting antibiotics and cold shock (Beckering et al., 2002; Salzberg et al., 2011; Wenzel et al., 2012). The *ytrGABCDEF* operon further codes for a putative ABC transporter, YtrBCDEF, which was suggested to be involved in acetoin utilization in *B. subtilis* (Quentin et al., 1999; Yoshida et al., 2000). Previous work already revealed that lack of YtrA leads to a competence defect and a decreased sporulation efficiency (Koo et al., 2017). In this work we could show that loss of genetic competence of the *ytrA* mutant is caused by the overexpression of the *ytrGABCDEF* operon. Furthermore, this phenotype seems to partially depend on the presence of the substrate binding protein YtrF. Surprisingly, transformations of a *ytrA* mutant also lacking both ATP-binding proteins, YtrB and YtrE, or both transmembrane proteins, YtrC and YtrD, only leads to a small number of colonies, suggesting that the loss of genetic competence of a *ytrA* mutant does not require the activity of the YtrBCDEF transporter.

Based on the partial complementation of genetic competence of the *ytrA* mutant upon *comKS* overexpression, one might expect that the loss of YtrA and the concomitant overexpression of the ABC transporter somehow interfere with a process upstream of ComK activation. However, competence is developed in an all or nothing scenario, and cells in which the ComK levels reach a certain threshold should become competent (Haijema et al., 2001; Maamar and Dubnau, 2005). Our observation that *comKS* overexpression only partially restores competence of the *ytrA* mutant suggests that ComK levels are not the only factor that limit competence of the *ytrA* mutant. If the *ytrA* deletion would only interfere with ComK activation, one would expect wild type-like competence upon overexpression of *comKS*, which was not the case. However, it is also possible that ComK levels in the *ytrA* mutant background are not sufficient to restore genetic competence, even after mannitol-induced overexpression of *comKS*. Another explanation could be that the activity of MecA is enhanced in the absence of YtrA leading to a faster degradation of ComK by the ClpCP complex. In addition to the loss of competence, the *ytrA* mutant produces a thicker cell wall as compared to the *B. subtilis* wild type, which could also have an impact on genetic competence. The DNA uptake apparatus must be adapted to cell wall thickness in order to ensure that the extracellular DNA can reach the ComG/ComE DNA transport complex. Due to the increased cell wall thickness upon overexpression of the YtrBCDEF ABC transporter, the DNA is probably unable to get in contact with the ComG pili. Overexpression of ComK will then result in the increased production of DNA-binding ComG on the cell surface of all cells of the population (compared to about 10% in the wild-type strain transformed with the classical two-step protocol). This would simply increase the probability that foreign DNA reaches the DNA uptake machinery in some cells, which then leads to the appearance of a few transformants as observed in our study. On the other hand, the results obtained by fluorescence microscopy revealed a decreased transcription from the ComK dependent *comG* promoter in the *ytrA* mutant. However, this expression is expected to be wild type-like if the action of the YtrBCDEF ABC transporter would not interfere with ComK activity and only block DNA uptake as a result of the thicker cell wall as suggested above. Again, the thicker cell wall might be responsible, since ComK expression is induced by the detection of extracellular quorum-sensing signals (both ComXPA and Rap-Phr systems) and this induction depends on the accessibility of the sensor domains for the pheromones [reviewed in Maier (2020)], which might be impaired in the strain with altered cell wall thickness.

In addition to the loss of genetic competence, it was previously shown that *ytrA* deletion leads to decreased sporulation efficiency (Koo et al., 2017) and we have shown that it also has a minor effect on biofilm formation. Considering the changed cell wall properties, this is in agreement with previous studies, which showed hampered biofilm formation upon disruption of cell wall biosynthesis (Bucher et al., 2015; Zhu et al., 2018). Taken together, we conclude that the overexpression of the YtrBCDEF ABC transporter upon deletion of *ytrA* leads to pleiotropic effects on alternative lifestyles of *B. subtilis* and to increased cell wall thickness, however, at the current stage it is unknown whether the proteins encoded in the *ytr* operon have a direct impact on competence, sporulation and biofilm formation or whether these pleiotropic effects are indirect consequences of the increased cell wall thickness.

Our results demonstrate that the YtrBCDEF ABC transporter is involved in the control of cell wall homeostasis, but it is not yet clear how this is achieved. An easy explanation would be that the system exports molecules necessary for cell wall synthesis, however, based on the presence of the substrate binding protein YtrF and on the critical role of this protein in preventing genetic competence, it can be assumed that the ABC transporter rather acts as an importer. YtrBCDEF could therefore be involved in the import of components required for the synthesis of peptidoglycan precursors or an unknown signal, which is involved in the regulation of peptidoglycan precursor production. In the latter case, overproduction of the ABC transporter YtrBCDEF would lead to an increased import of this unknown signal molecule and thus, to an enhanced peptidoglycan precursor synthesis leading to the production of a thicker cell wall. It is also possible that YtrBCDEF may not act as a transporter at all and simply modulate the activity of other enzymes that participate in cell wall metabolism. This could potentially explain why the *B. subtilis* strain lacking YtrA as well as the ATP-binding proteins, latter of which are usually essential for the activity of ABC transporters, still produce a thicker cell wall and remain non-competent. Strikingly, the C-terminus of YtrF contains a FtsX-like domain. In *B. subtilis*, the ABC transporter FtsEX activates the cell wall hydrolase CwlO *via* direct protein-protein interaction thereby affecting cell elongation (Meisner et al., 2013). We speculate that the FtsX-like domain of YtrF could thus be required for the interaction with other proteins.

Based on its expression pattern, the *ytr* operon was described as a reporter for glycopeptide antibiotics, such as vancomycin or ristocetin (Hutter et al., 2004) and antibiotics that interfere with the lipid II cycle, such as nisin (Wenzel et al., 2012). Whether this induction of *ytrGABCDEF* expression leads to an increased resistance toward those antibiotics is not clear, but recent results indicate that it has indeed an impact on nisin resistance (Senges et al., 2021). Interestingly, the substrate binding lipoprotein YtrF contains a MacB-like periplasmic core domain in its N-terminus. MacB proteins are usually involved in resistance toward antibiotics and peptide toxins (Greene et al., 2018), suggesting that YtrF could potentially bind antibiotics. It is tempting to speculate that cell wall-acting antibiotics serve as an exogenous signal to activate the expression of the ABC transporter YtrBCDEF, which leads to the production of a thicker cell wall and with this, to an increased resistance to these antibiotics. However, how this could still be achieved in the absence of the ATP-binding proteins YtrB and YtrE remains elusive. Future work will need to address the precise mechanism by which the YtrBCDEF ABC transporter affects cell wall synthesis in *B. subtilis*.

## Data Availability Statement

The original contributions presented in the study are included in the article/Supplementary Material, further inquiries can be directed to the corresponding author/s.

## Author Contributions

MB, JS, and JR: design of the study and writing the paper. MB and LS: experimental work. MB, LS, JR, and JS: data analysis. All authors contributed to the article and approved the submitted version.

## Conflict of Interest

The authors declare that the research was conducted in the absence of any commercial or financial relationships that could be construed as a potential conflict of interest.
