# Supplementary figures and images for: Influence of the ABC Transporter YtrBCDEF of Bacillus subtilis on Competence, Biofilm Formation and Cell Wall Thickness
Source: Front Microbiol. 2021 Apr 8;12:587035. doi: 10.3389/fmicb.2021.587035 (PMC8060467; doi:10.3389/fmicb.2021.587035)

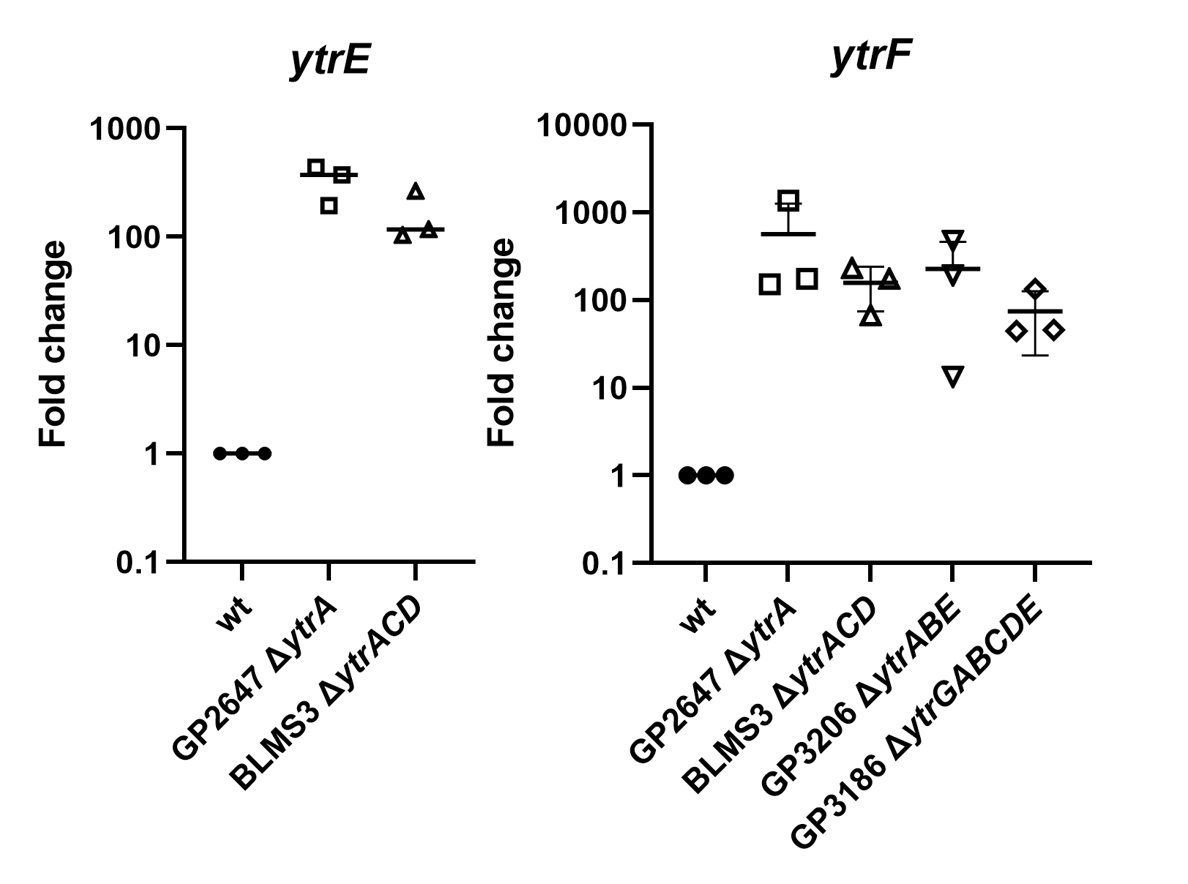

Supplement: Supplementary file 2 [file Image_1.TIF]
